# The Relationship between Big Five Personality Traits and Depression in the German-Speaking D-A-CH Region Including an Investigation of Potential Moderators and Mediators

**DOI:** 10.3390/ejihpe14080144

**Published:** 2024-07-30

**Authors:** Susanne Strohmaier, Manuel Pillai, Jakob Weitzer, Emilie Han, Lukas Zenk, Brenda M. Birmann, Martin Bertau, Guido Caniglia, Manfred D. Laubichler, Gerald Steiner, Eva S. Schernhammer

**Affiliations:** 1 Department of Epidemiology, Center for Public Health, Medical University of Vienna, 1090 Vienna, Austria; 2 Office of Crisis Management, Federal Chancellery of the Republic of Austria, Ballhausplatz 2, 1010 Vienna, Austria; 3 Department for Knowledge and Communication Management, Faculty of Business and Globalization, University of Continuing Education Krems, 3500 Krems, Austria; lukas.zenk@donau-uni.ac.at (L.Z.);; 4 Channing Division of Network Medicine, Department of Medicine, Brigham and Women’s Hospital and Harvard Medical School, Boston, MA 02115, USA; 5 Institute of Chemical Technology, Freiberg University of Mining and Technology, 09599 Freiberg, Germany; 6 Saxonian Academy of Sciences, Karl-Tauchnitz-Straße 1, 04107 Leipzig, Germany; 7 Fraunhofer-Institute for Ceramic Technologies and Systems IKTS, Fraunhofer Technology Center for High-Performance Materials THM, Am St.-Niclas-Schacht 13, 09599 Freiberg, Germany; 8 Konrad Lorenz Institute for Evolution and Cognition Research (KLI), 3400 Klosterneuburg, Austria; 9 School of Life Sciences, Arizona State University, Tempe, AZ 85281, USA; manfred.laubichler@asu.edu; 10 Santa Fe Institute, Santa Fe, NM 87501, USA; 11 Complexity Science Hub, 1080 Vienna, Austria; 12 Department of Epidemiology, Harvard T.H. Chan School of Public Health, Boston, MA 02115, USA

**Keywords:** big five personality traits, depression, mediation, optimism, empathy, D-A-CH region

## Abstract

Considerable evidence links the “Big Five” personality traits (neuroticism, extroversion, conscientiousness, agreeableness, and openness) with depression. However, potential mediating and moderating factors are less well understood. We utilized data from a cross-sectional survey of 3065 German-speaking adults from the D-A-CH region to estimate multivariable-adjusted odds ratios and 95% confidence intervalsbetween personality traits and lifetime prevalence of depression (overall and stratified by sex and age). We further explored proportions mediated by psychosocial factors optimism, empathy, perspective-taking, work–life balance, and interpersonal trust. High levels of neuroticism were associated with more than two-fold higher odds of depression, whereas higher levels of conscientiousness were associated with approximately 30% lower odds of depression. The association with neuroticism persisted in all investigated subgroups; apparently, stronger associations for females and participants aged ≥60 years did not correspond to statistically significant interactions. Overall and across all strata, the association of neuroticism with depression appeared to be mediated in part by the considered psychosocial factors; optimism explained the largest proportion of the association. Our results provide empirical evidence for the dynamic predisposition model. Further investigations of these relationships are warranted in longitudinal data with more precise outcome assessments.

## 1. Introduction

### 1.1. Depression Prevalence

Depression is incapacitating, costly, and highly prevalent and represents one of the leading causes of ill health and disability worldwide, making it a critical public health concern. Indeed, according to the Global Burden of Disease Study 0 in 2019—before the COVID-19 pandemic—approximately 280 million people were living with depression [1] corresponding to 3.6% of the population, with more women (170 million) than men (110 million) affected.

Reported prevalence estimates for Europe vary; the World Health Organization (WHO) [2] reported a prevalence of 4.4% based on data from the Global Burden of Disease study, whereas Arias-de la Torre et al. [3] reported 6.4% based on data from the European Health Interview Survey. For the countries of the D-A-CH region (Germany [D], Austria [A], and Switzerland [CH]) the WHO reports similar estimates for all three countries (5.1% Austria, 5.2% Germany, and 5.0% Switzerland), while Arias-de la Torre et al. reported quite different estimates for Austria (4.3%) and Germany (9.2%) but do not provide data on Switzerland. Both sources report higher prevalences among women and with increasing age.

### 1.2. Risk Factors for Depression

Given the public health burden of depression, there is a need to identify risk factors and targets for preventive as well as therapeutic measures. In addition to sex and age, several risk factors have been established including socio-demographic (e.g., low education, unstable employment, marital disruptions), behavioral (e.g., sedentary lifestyle, smoking), or biological (e.g., dysregulation of the hypothalamic–pituitary–adrenal [HPA] axis) factors [4].

However, in recent years, the long-standing theory that personality types are responsible for some forms of psychopathology received more attention in empirical [5,6,7] and therapeutic studies [8] on depression and on affective disorders more generally.

Personality has traditionally been understood as comprising two components: temperament, referring to the biologically rooted, early-established, and stable individual variations in emotion and its management, and character, referring to individual variations resulting from socialization processes. However, the delineations between these constructs have come into question given an emerging body of literature suggesting that personality traits exhibit all the attributes of temperament, such as genetic and biological foundations and considerable stability across the lifespan [9,10]. Consequently, the terms “personality” and “temperament” are now frequently used interchangeably [11].

### 1.3. Characterizing Personality

Numerous personality classifications have been put forward throughout the past century, but by the 1990s, they were consolidated into a consensus taxonomy known as the Five-Factor Model (FFM) [12]. The most dominant “Big Five” model suggests that personality manifests across five traits: neuroticism (easily upset, maladjusted, not calm), extroversion (assertive, energetic, talkative), conscientiousness (dependable, orderly, responsible), agreeableness (cooperative, good-natured, trusting), and openness (imaginative, independent-minded, intellectual) [13]. The Big Five personality traits are inferred through factor analysis; i.e., each trait represents a statistical common factor of several more specific personality aspects [14,15,16]. Consequently, personality can be explored at different levels within the personality hierarchy, ranging from broad high-order traits to more specific lower-order facets [16].

### 1.4. Relationship between Big Five Personality Traits and Depression

A vast body of literature reports on the association between Big Five personality traits and various (mental) health outcomes [17]. Three more recent meta-analyses focused on their associations with depressive symptoms [18,19,20] among other psychological outcomes [18]. The work by Kotov et al. 2010 [18] extensively covered the relationship between the Big Five traits and anxiety, major depressive disorder, and substance use disorder including up to 63 studies (depending on the outcome) and derived summary estimates for various outcomes. High levels of neuroticism and low levels of conscientiousness were reportedly linked to all considered outcomes. Agreeableness and openness were reported to be largely unrelated to the respective diagnoses. Hakulinen et al. [19] meta-analyzed 10 prospective cohort studies from Europe, Australia, and the United States and reported that low extroversion, high neuroticism, and low conscientiousness were associated with an increased risk of depressive symptoms during follow-up, with the most pronounced effects for neuroticism. The most recent thesis work by Chavoshi [20] investigated 243 studies that reported correlations between personality traits and depressive symptoms published between 2000 and 2022. The fairly large number of studies is owed to a rather broad definition of relevant outcome measures and their objective to also consider reported moderating factors such as sex and to consider various types of depression measures. Overall, they report a significant positive correlation with neuroticism and negative correlations with extroversion, conscientiousness, and openness.

In summary, all three recent meta-analyses consistently concluded that the most marked associations were reported between high levels of neuroticism and higher prevalence of depressive symptoms. There is also consistent evidence of associations between lower levels of conscientiousness and extroversion and depressive symptoms; however, there are some inconsistencies regarding the strength of these associations. Other than previous meta-analyses, Chavoshi [20] also reported a negative correlation between openness and depressive symptoms.

Interestingly, none of these meta-analyses included results based on data from the D-A-CH region. This might be because previous studies in the region mainly involved either clinical [21,22] or convenience samples [23,24] with mostly limited sample sizes or focused on other psychological outcomes like suicidality [25] or more general subjective well-being [26]. Hence there is a need for a larger scale, more comprehensive evaluation of these associations based on representative data from that region. To that end, we have data available from over 3000 participants in an online questionnaire who were quota-sampled to match the respective population distributions for gender, age, and region of residence. Participants provided information on their lifetime history of depression and personality profiles as well as several other relevant socioeconomic and psychosocial factors.

We expected to observe associations between high levels of neuroticism and a higher risk of lifetime occurrence of depression as well as between low levels of conscientiousness and extroversion and a higher risk of depression, as reported in previous literature.

### 1.5. Potential Mediating Factors

Despite providing estimates for the relationship between the Big Five traits and depression risk in the German-speaking D-A-CH region, it is also of interest to shed more light on the underlying mechanism of these associations. A particularly relevant question is the extent to which other psychosocial concepts, such as optimism, empathy, perspective-taking, interpersonal trust, and perceived work–life balance would mediate any such associations. There is empirical evidence that personality traits are linked to empathy [27,28], interpersonal trust [29,30], optimism [31], and work–life balance [32], which is one of the essential prerequisites to motivate a mediation analysis [33]. Furthermore, these psychosocial aspects are risk factors for depression [34,35,36,37]—another prerequisite for mediation analysis—however, with the caveat that personality traits might not have been considered as additional adjustment variables in these analyses.

Only a few studies have explicitly quantified the mediating role of these factors in the association between personality traits and depression. Lee [38] considered four empathy-related traits—personal distress, fantasy, empathic concern, and perspective-taking—in a hierarchical regression analysis motivated by previous evidence that these traits showed associations with neuroticism on the one hand and had been linked to depressive symptoms on the other hand. They concluded that empathic traits play a mediating role in the relationship between neuroticism and depression in a study among 204 American college students. Serrano et al. [39] studied the mediating role of optimism in the association between the Big Five traits and subjective well-being more generally. The conceptional underpinning of their analyses stemmed from a model given by Sharpe et al. [31] who suggested that an interplay between an affective, a social, and a persistent pathway describing some inherent characteristics of certain personality traits and in turn a personality profile characterized by low neuroticism, high extroversion, agreeableness, and conscientiousness would develop more optimistic beliefs about life event and hence more adaptive behaviors and improve mental health. They found that optimism was a substantial mediator in the relationship of extroversion and neuroticism with subjective well-being. We therefore hypothesize that any effects between neuroticism and extroversion and depression are at least partially mediated by the considered psychosocial factors, particularly empathy and optimism.

## 2. Materials and Methods

### 2.1. Study Sample

Data were collected via an online survey designed by a research team from the Medical University of Vienna and the University for Continuing Education Krems as well as other members of the Transatlantic research laboratory for complex societal challenges (https://www.donau-uni.ac.at/en/university/faculties/business-globalization/research/lab_complex-societal-challenges.html). The questionnaire consisted of 74 questions regarding lifestyle, health, and COVID-19-related mitigation measures, as well as several psychological instruments. The survey was implemented by the market research institute INTERROGARE, Bielefeld, Germany, between 21 July and 8 August 2021. Participants had to be 18 years of age or older, German-speaking, and residing in Germany, Austria, or Switzerland. The market research institute uses online panels, i.e., databases providing a broad general population coverage of potential participants. Recruitment is facilitated via “open enrollment” and “by invitation only” campaigns via email and online marketing channels. Potential participants are quota-sampled to match the respective population distributions for gender, age, and region of residence to strive for representativeness. For a detailed comparison regarding gender and age distribution by country, we refer to Supplementary Figures S1–S3 in [40]. Informed consent was implied when participants completed and submitted the survey. In total, 3067 adults completed the questionnaire; information on response rates and characteristics of non-responders is not available. Collected data did not include any information enabling participant identification and were only accessed and analyzed by the research team. Hence, the study was exempted by the Ethics Board of the Medical University of Vienna.

### 2.2. Personality Trait Assessment

The Big Five personality traits—neuroticism, extraversion, conscientiousness, openness, and agreeableness—were assessed using the German short version of the Big Five Inventory (BFI-S) developed by Gerlitz and Schupp [41] for the German Socio-Economic Panel Study (GSOEP). The BFI-S includes three items per dimension, and participants are asked to rate the respective three statements on a 7-point Likert scale ranging from 1 = “does not apply to me at all” to 7 = “applies to me perfectly”. The resulting score for each trait ranges from 3 to 21, with higher values indicating higher levels of a specific personality domain. The BFI-S has been validated in relation to the NEO Personality Inventory revised (NEO-PI-R) [13] in addition to showing acceptable levels of internal consistency, stability over 18 months, as well as discriminant validity [42].

### 2.3. Outcome Assessment

Participants were asked whether their physician had ever told them they had any of a list of 15 diseases, including depression. They were further given the option to enter any diagnosis in a free text or indicate that they had not been told about any of the listed diagnoses. Results based on the German Health Interview and Examination Surveys for Adults (DEGS1) indicated that 73.4% of participants indicating a clinician-diagnosed depression in fact met the criteria for any mental health disorder in the Composite International Diagnostic Interview (CIDI) [43]. These validity estimates are consistent with reports from other European populations [44,45,46].

### 2.4. Ascertainment of Covariates

Numerous sociodemographic variables were assessed including gender, age, country of residence (Austria, Germany, Switzerland), area of residence (rural vs. urban), ethnicity (Caucasian vs. non-Caucasian), migration history (first generation, second generation, none), marital status (single, married or in a relationship, divorced, widowed), level of attained education (middle school, apprenticeship, high school, university degree), monthly household income (in 10 categories ranging from less than 1000 Euro (or CHF) to more than 8000 Euro (or CHF), type of employment (full-time, part-time, currently without employment), and whether their work included working at night or not.

Additionally, information on several lifestyle-related behaviors was collected including smoking status (current, former, never), frequency of physical activity (how many days a week they engage in activity that raises breathing or heart rate for at least 10 min), body mass index (BMI), and estimates of work–life balance. The latter was enquired using the validated “Trierer Kurzskala zur Messung von Work–Life Balance” [47], which involves ranking 5 statements about work and private life on a scale from 1 (totally disagree) to 6 (totally agree), which leads to a total score ranging from 5 to 30 with a higher score indicating higher work–life balance.

In addition to sociodemographic and lifestyle variables, several personality characteristics were assessed: Empathy and perspective-taking were measured using a questionnaire (in German: Fragebogen fuer Empathie und Perspektivenuebernahme) consisting of nine statements related to behaviors indicating empathy or perspective-taking, respectively. Each of these 18 statements had to be rated on a six-point Likert scale (0 = never, 5 = all the time), which resulted in a total score from 0 to 45 for each trait, for which higher values indicate a more pronounced manifestation of the trait [48]. Optimism was assessed using the German version of the Life-Orientation Test revised (LOT-R) [49,50]. The six items pertaining to optimism and pessimism were ranked on a five-point Likert scale (1 = completely disagree, 5 = completely agree), leading to a total score between 6 and 30 with higher levels indicating higher levels of optimism. Interpersonal trust was measured using the validated “Kurzskala für interpersonales Vertrauen” (KUSIV3) [51] asking participants to rank 3 items on trust on a five-point Likert scale, which are then averaged. Higher averaged scores reflect higher levels of interpersonal trust.

### 2.5. Statistical Analysis

Participant characteristics for the total study population and separately for females and males were summarized as absolute and relative frequencies for categorical and as mean values and standard deviations (SD) for continuous variables. For each Big Five personality trait as well as the considered psychosocial factors empathy, perspective-taking, optimism, interpersonal trust, and work–life balance, we report McDonald’s omega as a measure of reliability for the underlying questionnaire.

Given the debate in current literature whether the associations between personality traits and depression were linear [11,52], we investigated the functional form of the potential association between the five personality traits as well as other continuous variables and depression by dividing the continuous variable into equal-width bins and then calculating and plotting the frequency of depression in each of these bins using the rms package in R [53]. These visualizations, followed by univariable models using restricted cubic splines with 4 knots (placed at the quintile cut-points of the distribution) and corresponding statistical tests, indicated non-linear functional forms for all personality traits except conscientiousness as well as for age, BMI, empathy, perspective-taking, and optimism. For ease of presentation and readability, we therefore decided to report odds ratios (ORs) and 95% confidence intervals (CIs) for the association between self-reported physician-diagnosed depression and each personality trait comparing participants with high vs. normal values of each personality trait. As suggested by Gerlitz and Schupp [41], values of 15 and above were considered as high, while values below 15 were considered normal. All other continuous variables showing indications for non-linearity were included using splines. Particularly, we fitted age- and gender-adjusted models including one personality trait at a time (Model 1) and models mutually adjusted for all personality traits (Model 2). In Model 3, we additionally adjusted for several socioeconomic characteristics including marital status, income, education, employment status, night shift work, country of residence, migration history, and ethnicity as well as the lifestyle-related variables physical exercise, smoking status, and BMI. As a first step to investigate the role of the additional psychosocial variables, we fitted a separate model additionally adjusting for optimism, empathy, perspective-taking, interpersonal trust, and work–life balance (Model 4).

If the total effect of a personality trait on depression risk remained statistically significant after adjustment for several risk factors (Model 3), we further quantified the proportion mediated and corresponding 95% CIs for each of the considered psychosocial concepts based on the approach described and implemented by Imai et al. [54]. We applied a linear model for the mediator–exposure relationship and a logistic regression model for the outcome including both the exposure and the mediator. The obtained regression coefficients for the exposure–mediator, exposure–outcome, and mediator–outcome relationship were combined as described in [54]. Additionally, we applied a heuristic approach to estimate the proportion mediated jointly by all considered covariates along the lines of Nevo et al. [55] comparing the regression coefficients for the exposure–outcome relationship in the model with (Model 4) and without (Model 3) the mediators. Corresponding 95% CIs were based on 500 bootstrap samples.

Since previous studies reported effect modification by gender and age [7], we stratified our analyses by gender and age in groups (18–39 years, 40–59 years, and 60 years and older) and tested for possible interactions utilizing a likelihood ratio test with appropriate degrees of freedom.

In sensitivity analyses, we also considered the personality traits as continuous variables: (i) assuming a linear relationship on the log odds scale as well as (ii) allowing for more flexibility in the functional form by using restricted cubic splines with 4 knots placed at the quintile cut-points of the distributions. All analyses were performed using R version 4.3.2.

## 3. Results

Among the 3067 participants included in our study, two participants identified as “gender diverse”, 1567 as female and 1498 as male. We restricted our analyses to the 3065 participants identifying as male or female for statistical reasons. Among these, 507 (16.5%) (201 males, 306 females) reported a previous depression diagnosis.

The average age was 47.98 (SD = 16.49) years, with males being older on average (mean = 51.95, SD = 16.02 years) than females (mean = 44.19, SD = 16.03 years) (see Table 1). Comparing socioeconomic characteristics among males and females, we observed that a higher proportion of male than female participants lived in urban areas, had a higher level of education, were full-time employed, were married or in a relationship, and were in the higher income category. The proportion of former or current smokers as well as the mean BMI and work–life balance score was also higher among males. Regarding psychosocial characteristics, mean scores of empathy and perspective-taking were higher in females, while males showed slightly higher mean scores of interpersonal trust and optimism. Obtained McDonald’s omega estimates for all personality traits except agreeableness with 0.60 were higher than 0.70 in our sample, which is considered acceptable [56]. For the remaining psychosocial factors, we obtained omega estimates between 0.79 for interpersonal trust and 0.93 for empathy as well as perspective-taking.

Considering the entire study population, we found associations between high levels of neuroticism and higher odds of depression and between high levels of conscientiousness and lower odds of depression, but no further significant associations for any other personality trait (Table 2). Comparing the results across different models, we observed the influence of the adjustment variables based on changes in the magnitude of ORs between Models 1 and 3; however, all associations consistently pointed in the same direction across models. We therefore consider the results based on Model 3 as the estimates of risk factor-adjusted total effects of a given personality trait with depression history. The change in estimates for neuroticism between Model 3 and Model 4 is however of particular interest, as the drop in effect size (from OR = 3.59; 95% CI = 2.87–4.48 to OR = 2.18; 95% CI = 1.70–2.81) provides evidence that at least part of the association of neuroticism with depression history is mediated by the additionally considered psychosocial characteristics (optimism, empathy, perspective-taking, interpersonal trust, and work–life balance).

Considering contributions to the covariate-adjusted association of neuroticism with depression history separately, estimated proportions mediated were 19.2% (95% CI = 13.6–27.0) for optimism, 10.7% (95% CI = 6.3–15.1) for empathy, 0.8% (95% CI = −0.25–2.0) for perspective-taking, 5.3% (95% CI = 2.7–9.0) for interpersonal trust, and 9.4% (95% CI = 5.4–13.0) for work–life balance. Heuristically, considering all mediators jointly gave a proportion mediated of 38.5% (95% CI = 30.7–49.0) (see Table 3).

When stratifying by gender, the association for high levels of neuroticism was comparable in both genders. Notably, we again observed drops in effect estimates between Models 3 and 4, which suggested that part of the association of neuroticism with depression is mediated by the additionally adjusted psychosocial factors. Interestingly, the proportion mediated by empathy seems to be higher in women (15.2% [9.8–24.4]) than in men (6.8% [2.1–14.0]), while the proportion mediated by optimism appears to be higher in males (21.6% [11.5–35.2]) than in females (17.7% [8.7–27.0]).

The sex-stratified analyses also yielded some apparently sex-specific associations for other personality traits (Table 4). These included a significant association of high levels of extroversion with lower odds of depression (OR = 0.60, 95% CI = 0.40–0.89) in men, but not in women (OR = 0.94, 95% CI = 0.69–1.28). For high levels of openness, on the other hand, we observed an increased risk of depression (OR = 1.55, 95% CI = 1.09–2.20) among males, but no significant association among females (OR = 1.07, 95% CI = 0.80–1.43). An association with low conscientiousness was more pronounced and statistically significant in women (OR = 0.59, 95% CI = 0.43–0.80); in men, the OR pointed in the same direction but was not significantly reduced (OR = 0.82, 95% CI = 0.57–1.18). We observed no significant association between agreeableness and depression in any sex-specific strata. Furthermore, none of the apparently sex-specific associations corresponded to a statistically significant interaction.

When stratifying by age (Table 5), we consistently observed an association between high levels of neuroticism and higher odds of depression across all age groups. Estimates based on Model 3 ranged from OR = 2.94 (95% CI = 2.02–4.30) in 18- to 39-year olds and OR = 3.67 (95% CI = 2.60–5.16) in 40- to 59-year olds to OR = 5.75 (95% CI = 3.45–9.59) in people aged 60 years and older. The proportion mediated by jointly considering all variables was, however, highest among those aged 18–39 years (46.1%) and lowest among the 60+ years subgroup (19.1%). The proportion mediated by the individual psychosocial factors displayed similar patterns, with the most pronounced change for empathy (proportions mediated were 19.7% for those aged 18–39 years and 3.7% for those aged 60+ years). Even though all estimates for the association with higher levels of conscientiousness were lower than one, the effect was most pronounced and significant for the age 60+ years stratum (OR = 0.42, 95% CI = 0.25–0.69). However, tests for effect modification were not significant. No further statistically significant age-specific associations or suggestive variation by age group were observed; only estimated ORs for agreeableness showed some fluctuation by age group.

Considering the personality traits as continuous variables, we qualitatively observed largely the same results (Supplemental Tables S1–S3). The results using a more flexible modeling approach using restricted cubic splines to capture potential non-linear effects are depicted in Supplementary Figures S3 and S4. The associations of neuroticism and agreeableness with depression suggested non-linearity, while the functional forms for the remaining personality traits appeared fairly linear.

## 4. Discussion

### 4.1. Summary of Findings

In our study based on 3065 participants in the German-speaking D-A-CH region, we found that high levels of neuroticism are associated with higher odds of depression, whereas higher levels of conscientiousness were associated with lower odds of depression.

Higher levels of neuroticism were associated with higher depression odds in all investigated subgroups. Effect estimates were slightly higher among females than among males. In age-stratified analyses, the highest ORs were observed among participants 60 years and older. However, none of the tests for interaction were significant. The observed lower odds associated with higher levels of conscientiousness seemed mainly to be driven by female participants and those aged 60 years and older. Overall and across all strata, we found indication that part of the association of neuroticism with depression was mediated by several psychosocial factors with optimism explaining the largest proportion of the mediated association.

### 4.2. Conceptional Models for the Observed Associations between Personality Traits and Depression

While our study had a cross-sectional design, our underlying assumption was that personality traits would be evident prior to the development of depression. This notion would in fact be compatible with several conceptional models summarized by Klein et al. [11], particularly the “precursor” and “predisposition” models. The precursor model assumes that personality and depression have shared etiological influences but do not have a direct causal influence, while the predisposition models suggest that the mechanisms underlying personality are distinct from those contributing to depression. Thus, the predisposition model implies a more nuanced interplay among risk factors and personality facets involving moderation and/or mediation—in line with the analysis approach utilized in our study.

### 4.3. Comparison to Literature–Overall

Our overall results regarding neuroticism are in line with previous literature, which consistently reported the strongest association between a higher risk for depression and a stronger manifestation of neuroticism [18,19,20]. However, effect sizes are difficult to compare given the differences in approaches, study design, and reported effect measures as well as variation in depression prevalence. Likewise, our results for conscientiousness are in line with results reported in meta-analyses [18,19]. The effect of extroversion did not reach statistical significance in our analysis but pointed in the same direction as previously reported, with higher levels of extroversion being associated with a lower risk of depression.

### 4.4. Mediation by Psychosocial Factors

Few previous studies thus far have empirically explored the mechanisms that potentially link personality traits to health outcomes. Not surprisingly, the few existing studies focused on neuroticism in relation to depression and subjective well-being more generally [38,39] and considered empathic traits and optimism as potential mediators. Similar to our study, Serrano et al. [39] reported that a considerable proportion of the effect of emotional stability (low neuroticism) on subjective well-being was mediated by optimism. This aligns with the viewpoint advocated by Dweck [57], who underscore the pivotal role of individuals’ fundamental beliefs, including those concerning anticipated future outcomes, in connecting personality traits with consistent behavioral patterns and experiences leading to depression. Lee [38] investigated the contribution of four types of empathic emotions (personal distress, fantasy, empathic concern, and perspective-taking) to the relationship between neuroticism and depression. Their results support that empathic traits play a role in the neuroticism–depression process. The biggest influence was attributed to fantasy, which was not assessed in the current study, while empathic concern and perspective-taking did not contribute significantly. Our results regarding perspective-taking are in line with this previous report. We do, however, find some evidence for mediation via empathic concern. These reported inconsistencies could be due to differences in assessing empathy dimensions and personality traits as well as the chosen analysis approach.

We found that work–life balance explained a proportion of the neuroticism–depression association comparable to that explained by empathy. Previous studies have linked neuroticism to greater conflict in balancing work and family demands [32], while poor work–life balance has in turn been linked to increased risk for health problems [58] and for depression in particular (e.g., [37]). Also, several authors reported considerable mediation by Cohen’s perceived stress scale [59], albeit using different assessments for neuroticism and depression [60,61]. However, our presented results warrant further confirmation in other samples and populations, ideally within a prospective study context.

Similarly, our consideration of interpersonal trust as a mediator was motivated by empirical evidence for exposure–outcome [19], exposure–mediator [30], and mediator–outcome [35] relationships. However, the observed effects are rather small (5% on average) and not significantly different from zero in some subgroups, which again calls for more comprehensive data to clarify this relationship.

When interpreting these results, one needs to keep in mind that the cross-sectional nature of our available data precludes the crucial assumption of a clear temporal ordering between exposure, mediator(s), and outcome. Furthermore, following modern causal inference theory, several additional untestable assumptions would need to be made [52] that become even more crucial when seeking to disentangle the contribution of one of several potential mediators to the overall effects [62]. Indeed, it is only under very restrictive assumptions that the individual proportions mediated would add up exactly to the proportion mediated jointly by all mediators.

### 4.5. Gender-Stratified Results

Previous studies have identified differences in these associations between men and women [7,63]. We did not observe any evidence for significant effect modification by gender, although the association for conscientiousness in the overall sample seems to be mainly driven by females. Furthermore, the association for extroversion for males is in line with previous literature, but not the observed association for females. Goodwin and Gotlib [64] also studied the interplay between gender and personality traits in relation to depression risk. While they worked under different assumptions regarding the role of these factors, they reported higher levels of neuroticism scores as well as higher depression prevalence among females as can also be observed in our sample (Table 3 and Supplementary Table S1). Certainly, the factors contributing to these suggestive gender differences deserve further investigation; Goodwin and Gotlib suggest societal influences and expectations as possible explanations. Our mediation analyses for neuroticism revealed that the contribution of other psychosocial factors, particularly empathy, varied considerably between sexes, which suggests a complex interplay between various personality characteristics.

### 4.6. Age-Stratified Results

There were several investigations regarding potential changes in personality traits across the lifespan [65,66] as well as investigations of the personality trait—depression relationship in the elderly [67,68]. However, only a few studies explicitly studied the effect modification of these associations by age. As in the study by Zhao et al. [7], our tests for effect modification were not significant; nonetheless, we observed that effect estimates, and especially those for neuroticism and conscientiousness, varied quite substantially by age and were most pronounced for participants aged 60 years and above. Furthermore, results from mediation analyses focusing on neuroticism indicated substantial differences in the proportion mediated by the psychosocial factors, both jointly and individually. Taken together, these results provide evidence that neither the Big Five personality traits nor other aspects of personality can be assumed to be entirely stable across the lifespan, as described by Roberts et al. [65,69], and that the mechanism(s) linking personality and depression are of a more complex and dynamic nature. The latter notion is captured in the dynamic predisposition model proposed by Ormel et al. [70]. However, our observations regarding apparent age dynamics have to be interpreted with caution given the cross-sectional design of our study.

### 4.7. Strengths and Limitations

Our investigations are based on a large dataset collected via an online survey that included a wide range of relevant socio-demographic and psychosocial variables and sampled participants to be representative of the German-speaking population of the three D-A-CH countries. We used validated instruments to assess personality traits and several other psychosocial constructs. Our sophisticated statistical approach addressed several shortcomings described in previous reports, e.g., our investigation of potentially non-linear associations. Furthermore, we used state-of-the-art mediation analysis techniques to shed light on the underlying mechanisms, particularly those linking neuroticism and depression.

Nevertheless, some limitations need to be acknowledged. While mainly validated tools were used, the self-reported nature could have introduced some degree of misclassification. This pertains also to our primary outcome—self-report of physician-diagnosed depression. However, previous studies in the geographic region using such measurements report sufficient accuracy and reliability [43]. The cross-sectional design of our survey hampered a thorough investigation of the time dynamics of some of the addressed associational links. This is particularly relevant to our considered mediation analyses, as it is conceivable that some of the considered psychosocial factors have been affected by a previous depressive episode, e.g., an individual who had previously suffered from depression worked to improve their work–life balance (and report their current rather than pre-depression state). Furthermore, it is conceivable that a state of emotional instability potentially due to depression at the time of the questionnaire return might influence the reporting of personality trait-related items. Hence, we want to stress that the presented mediation analysis results serve a more exploratory purpose, and future longitudinal studies are needed to confirm these results. Lastly, while the sampling design of the study was chosen to improve representativeness, the study was conducted among the German-speaking population of the D-A-CH region in 2021 during the COVID-19 pandemic; therefore, our presented results might not be directly generalizable to other populations.

## 5. Conclusions

In summary, we confirmed previously reported associations between some Big Five personality traits and depression in the German-speaking D-A-CH region based on a representative sample using a thoughtful statistical approach. Furthermore, our exploratory mediation analysis suggests new insights regarding potential mediating factors in those associations. Collectively, our findings provide some empirical evidence for the conceptional dynamic predisposition model put forward by Ormel et al. [70]. Nevertheless, these relationships warrant further investigation in longitudinal data with more precise outcome assessments and under different societal circumstances.

## Figures and Tables

**Table 1:** Characteristics of the study population (N = 3065) overall and stratified by gender.

Variables	Females (N = 1567)	Males (N = 1498)	Overall (N = 3065)
Age ^a^	44.19 (16.03)	51.95 (16.02)	47.98 (16.49)
Empathy ^a^	31.18 (8.01)	27.90 (8.34)	29.57 (8.33)
Perspective-taking	28.88 (7.61)	27.42 (8.07)	28.17 (7.87)
Interpersonal trust score ^a^	3.07 (0.87)	3.18 (0.87)	3.12 (0.87)
Optimism score ^a^	19.88 (4.45)	20.14 (4.15)	20.01 (4.31)
Work–life balance ^a^	20.91 (5.53)	21.58 (5.50)	21.24 (5.52)
Ethnicity ^b^			
Caucasian	1404 (89.6)	1400 (93.5)	2804 (91.5)
Other	163 (10.4)	98 (6.5)	261 (8.5)
Migration history ^b^			
First generation	431 (27.5)	401 (26.8)	832 (27.1)
Second generation	161 (10.3)	136 (9.1)	297 (9.7)
No migration background	975 (62.2)	961 (64.2)	1936 (63.2)
Country of residence ^b^			
Austria	521 (33.2)	498 (33.2)	1019 (33.2)
Germany	531 (33.9)	492 (32.8)	1023 (33.4)
Switzerland	515 (32.9)	508 (33.9)	1023 (33.4)
Area of residence ^b^			
Urban	789 (50.4)	867(57.9)	1656 (54.0)
Rural	778 (49.6)	631 (42.1)	1409 (46.0)
Education ^b^			
Middle school	150 (9.6)	71 (4.7)	221 (7.2)
Apprenticeship	698 (44.5)	667 (44.5)	1365 (44.5)
High school diploma	374 (23.9)	376 (25.1)	750 (24.5)
University degree	345 (22.0)	384 (25.6)	729 (23.8)
Marital status ^b^			
Single	503 (32.1)	410 (27.4)	913 (29.8)
Married or in a relationship	822 (52.5)	906 (60.5)	1728 (56.4)
Divorced	185 (11.8)	148 (9.9)	333 (10.9)
Widowed	57 (3.6)	34 (2.3)	91 (3.0)
Smoking status ^b^			
Never	697 (44.5)	585 (39.1)	1282 (41.8)
Former	386 (24.6)	453 (30.2)	839 (27.4)
Current	484 (30.9)	460 (30.7)	944 (30.8)
Exercise/week ^a^	2.88 (2.34)	3.07 (2.35)	2.98 (2.35)
Body mass index	25.15 (5.84)	26.99 (5.00)	26.05 (5.52)
Employment ^b^			
Full-time	457 (29.2)	611 (40.8)	1068 (34.8)
Part-time	251 (16.0)	98 (6.5)	349 (11.4)
Currently without employment	643 (41.0)	577 (38.5)	1220 (39.8)
Missing	216 (13.8)	211 (14.2)	428 (14.0)
Income			
Low	931 (59.4)	680 (45.4)	1611 (52.6)
Medium	403 (25.7)	485 (32.4)	888 (29.0)
High	233 (14.9)	333 (22.2)	566 (18.5)
Night shift work ^b^	13 (0.8)	21 (1.4)	34 (1.1)

^a^ Mean ± SD (all such values), ^b^ N (percentages) (all such values).

**Table 2:** Odds ratios (ORs) and 95% confidence intervals (CIs) for lifetime history of depression comparing high vs. normal manifestations of the Big Five personality traits among the N = 3065 participants of the DACH survey.

		Model 1 ^1^	Model 2 ^2^	Model 3 ^3^	Model 4 ^4^
	Cases/N	OR (95% CI)	OR (95% CI)	OR (95% CI)	OR (95% CI)
Neuroticism					
Normal	283/2426	1 (ref)	1 (ref)	1 (ref)	1 (ref)
High	224/639	3.99 (3.22–4.93) *	3.96 (3.19–4.90) *	3.59 (2.87–4.48) *	2.18 (1.70–2.81) *
Extroversion					
Normal	350/1995	1 (ref)	1 (ref)	1 (ref)	1 (ref)
High	157/1070	0.76 (0.62–0.94) *	0.85 (0.68–1.08)	0.80 (0.63–1.02)	0.79 (0.61–1.01)
Openness					
Normal	270/1727	1 (ref)	1 (ref)	1 (ref)	1 (ref)
High	237/1338	1.13 (0.93–1.37)	1.22 (0.99–1.51)	1.22 (0.98–1.52)	1.11 (0.87–1.40)
Agreeableness					
Normal	257/1447	1 (ref)	1 (ref)	1 (ref)	1 (ref)
High	250/1608	0.83 (0.68–1.01)	0.93 (0.75–1.15)	0.98 (0.78–1.22)	0.88 (0.68–1.12)
Conscientiousness					
Normal	210/1067	1 (ref)	1 (ref)	1 (ref)	1 (ref)
High	297/1998	0.66 (0.54–0.81) *	0.64 (0.51–0.81) *	0.69 (0.54–0.87) *	0.68 (0.53–0.87) *

^1^ Model 1 adjusted for gender and age (using restricted cubic splines with 5 knots). ^2^ Model 2 additionally adjusted for all other Big Five personality traits. ^3^ Model 3 additionally adjusted for country of residence (Austria, Germany, Switzerland), migration history (first generation, second generation, no migration history), marital status (single, married or in a relationship, divorced, widowed), income (continuous), education (middle school, apprenticeship, high school, university degree), ethnicity (Caucasian, other), BMI (using restricted cubic splines with 4 knots), exercise (number of exercise units/week), smoking status (never, former, current), employment (full-time, part-time, not currently employed, missing), night shift work (yes, no). ^4^ Model 4 additionally adjusted for empathy score (restricted cubic spline with 3 knots), perspective-taking (restricted cubic spline with 3 knots), optimism score (restricted cubic spline with 3 knots), work–life balance score, interpersonal trust score. * indicates statistically significant.

**Table 3:** Proportion-mediated and 95% confidence intervals for individual psychosocial factors and all factors considered jointly.

Analysis Sets	Optimism ^a^	Empathy ^a^	Perspective-Taking ^a^	Interpersonal Trust ^a^	Work–Life Balance ^a^	Jointly Considering All Psychosocial Factors ^b^
Overall	19.2% (13.6–27.0)	10.7% (6.3–15.1)	0.8% (−0.25–2.0)	5.3% (2.1–9.0)	9.4% (5.4–13.0)	38.8% (30.7–49.0)
Males	21.6% (11.5–35.2)	6.8% (2.1–14.0)	0.2% (−0.7–2.0)	5.9% (0.0–13.0)	9.4% (3.6–18.1)	42.1% (27.0–64.8)
Females	17.7% (8.7–27.0)	15.2% (9.8–24.4)	1.3% (−0.2–4.2)	4.8% (0.9–10.1)	9.9% (3.5–15.3)	35.5% (26.4–48.5)
Age 18–39 yrs	23.8% (12.3–41.1)	19.7% (9.2–34.3)	3.1% (−0.2–9.1)	4.1% (−1.1–11.2)	9.7% (3.4–19.2)	46.1% (30.1–70.2)
Age 40–59 yrs	19.4% (8.4–29.5)	11.9% (7.0–20.1)	0.8% (−0.3–3.3)	4.3% (−2.0–10.1)	10% (3.5–18.0)	47.2% (31.8–66.4)
Age 60+ yrs	11.8% (2.3–28.3)	3.7% (4.2–9.1)	0.7% (−4.1–1.7)	5.0% (1.2–11.3)	3.0% (−2.3–12.1)	19.1% (1.4–33.2)

^a^ Estimated using the approach suggested by Imai et al. (2010). ^b^ Heuristically estimated following Nevo et al. (2017); confidence intervals are based on n = 500 bootstrap samples.

**Table 4:** Odds ratios (ORs) and 95% confidence intervals (CIs) for lifetime history of depression comparing high vs. normal manifestations of the Big Five personality traits among the N = 3065 participants of the DACH survey stratified by gender.

		Model 1 ^1^	Model 2 ^2^	Model 3 ^3^	Model 4 ^4^	p-Interaction
	Cases/N	OR (95% CI)	OR (95% CI)	OR (95% CI)	OR (95% CI)	
MALES						
Neuroticism						
Normal	132/1288	1 (ref)	1 (ref)	1 (ref)	1 (ref)	
High	69/210	4.14 (2.92–5.88) *	4.04 (2.84–5.74) *	3.42 (2.34–4.99) *	2.04 (1.33–3.12) *	
Extroversion						
Normal	151/1016	1 (ref)	1 (ref)	1 (ref)	1 (ref)	
High	50/482	0.66 (0.47–0.94) *	0.67 (0.46–0.98) *	0.60 (0.40–0.89) *	0.54 (0.35–0.83) *	
Openness						
Normal	109/875	1 (ref)	1 (ref)	1 (ref)	1 (ref)	
High	92/623	1.23 (0.91–1.66)	1.44 (1.04–2.00)	1.55 (1.09–2.20)	1.35 (0.93–1.97)	
Agreeableness						
Normal	110/768	1 (ref)	1 (ref)	1 (ref)	1 (ref)	
High	91/730	0.88 (0.65–1.19)	1.04 (0.74–1.45)	1.10 (0.77–1.57)	1.07 (0.72–1.59)	
Conscientiousness						
Normal	86/559	1 (ref)	1 (ref)	1 (ref)	1 (ref)	
High	115/939	0.76 (0.55–1.03)	0.78 (0.55–1.10)	0.82 (0.57–1.18)	0.85 (0.58–1.25)	
FEMALES						
Neuroticism						
Normal	151/1138	1 (ref)	1 (ref)	1 (ref)	1 (ref)	
High	155/429	3.86 (2.96–5.04) *	4.00 (3.05–5.24) *	3.83 (2.89–5.08) *	2.38 (1.72–3.29) *	0.82
Extroversion						
Normal	199/979	1 (ref)	1 (ref)	1 (ref)	1 (ref)	
High	107/588	0.84 (0.65–1.10)	1.01 (0.75–1.35)	0.94 (0.69–1.28)	0.94 (0.68–1.29)	0.37
Openness						
Normal	161/852	1 (ref)	1 (ref)	1 (ref)	1 (ref)	
High	145/715	1.07 (0.84–1.38)	1.10 (0.83–1.45)	1.07 (0.80–1.43)	0.96 (0.70–1.31)	0.22
Agreeableness						
Normal	147/689	1 (ref)	1 (ref)	1 (ref)	1 (ref)	
High	159/868	0.80 (0.62–1.03)	0.88 (0.66–1.17)	0.91 (0.68–1.22)	0.78 (0.56–1.08)	0.21
Conscientiousness						
Normal	124/508	1 (ref)	1 (ref)	1 (ref)	1 (ref)	
High	182/1059	0.60 (0.46–0.78) *	0.56 (0.41–0.75) *	0.59 (0.43–0.80) *	0.56 (0.40–0.78) *	0.09

^1^ Model 1 adjusted for gender and age (using restricted cubic splines with 5 knots). ^2^ Model 2 additionally adjusted for all other Big Five personality traits. ^3^ Model 3 additionally adjusted for country of residence (Austria, Germany, Switzerland), migration history (first generation, second generation, no migration history), marital status (single, married or in a relationship, divorced, widowed), income (continuous), education (middle school, apprenticeship, high school, university degree), ethnicity (Caucasian, other), BMI (using restricted cubic splines with 4 knots), exercise (number of exercise units/week), smoking status (never, former, current), employment (full-time, part-time, not currently employed, missing), night shift work (yes, no). ^4^ Model 4 additionally adjusted for empathy score (restricted cubic spline with 3 knots), perspective-taking (restricted cubic spline with 3 knots), optimism score (restricted cubic spline with 3 knots), work–life balance score, interpersonal trust score. * indicates statistically significant.

**Table 5:** Odds ratios (ORs) and 95% confidence intervals (CIs) for lifetime history of depression comparing high vs. normal manifestations of the Big Five personality traits among the N = 3065 participants of the DACH survey stratified by age.

		Model 1 ^1^	Model 2 ^2^	Model 3 ^3^	Model 4 ^4^	p-Interaction
	Cases/N	OR (95% CI)	OR (95% CI)	OR (95% CI)	OR (95% CI)	
AGED 18–39 YEARS						
Neuroticism						
Normal	79/864	1 (ref)	1 (ref)	1 (ref)	1 (ref)	
High	79/158	3.14 (2.21–4.48) *	3.07 (2.15–4.38) *	2.94 (2.02–4.30) *	1.77 (1.16–2.70) *	
Extroversion						
Normal	120/714	1 (ref)	1 (ref)	1 (ref)	1 (ref)	
High	38/308	0.70 (0.47–1.03)	0.81 (0.53–1.24)	0.69 (0.44–1.09)	0.68 (0.42–1.10)	
Openness						
Normal	84/568	1 (ref)	1 (ref)	1 (ref)	1 (ref)	
High	74/454	1.10 (0.78–1.55)	1.21 (0.83–1.75)	1.24 (0.84–1.83)	1.08 (0.71–1.66)	
Agreeableness						
Normal	93/561	1 (ref)	1 (ref)	1 (ref)	1 (ref)	
High	65/461	0.79 (0.60–1.12)	0.90 (0.61–1.32)	0.99 (0.66–1.48)	0.91 (0.58–1.43)	
Conscientiousness						
Normal	87/485	1 (ref)	1 (ref)	1 (ref)	1 (ref)	
High	71/537	0.66 (0.47–0.93)	0.68 (0.46–1.01)	0.74 (0.49–1.11)	0.77 (0.50–1.19)	
AGED 40–59 YEARS						
Neuroticism						
Normal	135/891	1 (ref)	1 (ref)	1 (ref)	1 (ref)	
High	105/247	4.04 (2.95–5.54) *	4.04 (2.95–5.54) *	3.67 (2.60–5.16) *	1.98 (1.32–2.98) *	
Extroversion						
Normal	158/727	1 (ref)	1 (ref)	1 (ref)	1 (ref)	
High	82/411	0.84 (0.62–1.14)	0.91 (0.64–1.27)	0.88 (0.61–1.27)	0.86 (0.58–1.26)	
Openness						
Normal	127/633	1 (ref)	1 (ref)	1 (ref)	1 (ref)	
High	113/505	1.13 (0.85–1.50)	1.18 (0.86–1.63)	1.12 (0.79–1.58)	1.05 (0.73–1.52)	
Agreeableness						
Normal	120/533	1 (ref)	1 (ref)	1 (ref)	1 (ref)	
High	120/605	0.82 (0.61–1.09)	0.85 (0.62–1.18)	0.87 (0.62–1.22)	0.73 (0.50–1.07)	
Conscientiousness						
Normal	83/349	1 (ref)	1 (ref)	1 (ref)	1 (ref)	
High	157/789	0.76 (0.56–1.03)	0.77 (0.54–1.08)	0.81 (0.56–1.17)	0.82 (0.55–1.21)	
AGED 60+ YEARS						
Neuroticism						
Normal	69/792	1 (ref)	1 (ref)	1 (ref)	1 (ref)	
High	40/113	5.34 (3.35–8.50) *	5.54 (3.44–8.91) *	5.75 (3.45–9.59) *	4.12 (2.33–7.29) *	0.17
Extroversion						
Normal	72/554	1 (ref)	1 (ref)	1 (ref)	1 (ref)	
High	37/351	0.77 (0.50–1.18)	0.89 (0.56–1.40)	0.80 (0.50–1.30)	0.82 (0.49–1.35)	0.88
Openness						
Normal	59/526	1 (ref)	1 (ref)	1 (ref)	1 (ref)	
High	50/379	1.20 (0.80–1.80)	1.35 (0.87–2.10)	1.32 (0.83–2.10)	1.15 (0.70–1.91)	0.86
Agreeableness						
Normal	44/363	1 (ref)	1 (ref)	1 (ref)	1 (ref)	
High	65/542	0.92 (0.61–1.39)	1.14 (0.73–1.80)	1.20 (0.75–1.93)	1.09 (0.65–1.85)	0.79
Conscientiousness						
Normal	40/233	1 (ref)	1 (ref)	1 (ref)	1 (ref)	
High	69/672	0.52 (0.34–0.79) *	0.44 (0.28–0.71) *	0.42 (0.25–0.69) *	0.40 (0.24–0.67) *	0.43

^1^ Model 1 adjusted for gender and age (using restricted cubic splines with 5 knots). ^2^ Model 2 additionally adjusted for all other Big Five personality traits. ^3^ Model 3 additionally adjusted for country of residence (Austria, Germany, Switzerland), migration history (first generation, second generation, no migration history), marital status (single, married or in a relationship, divorced, widowed), income (continuous), education (middle school, apprenticeship, high school, university degree), ethnicity (Caucasian, other), BMI (using restricted cubic splines with 4 knots), exercise (number of exercise units/week), smoking status (never, former, current), employment (full-time, part-time, not currently employed, missing), night shift work (yes, no). ^4^ Model 4 additionally adjusted for empathy score (restricted cubic spline with 3 knots), perspective-taking (restricted cubic spline with 3 knots), optimism score (restricted cubic spline with 3 knots), work–life balance score, interpersonal trust score. * indicates statistically significant.

## Data Availability

The raw data supporting the conclusions of this article can be made available upon reasonable request by the corresponding author.

## Supplementary Materials

The following supporting information can be downloaded at https://www.mdpi.com/article/10.3390/ejihpe14080144/s1, Supplemental Table S1: Distribution of Big Five personality among the entire study population as well as gender and age strata. Supplemental Table S2: Odds ratios (ORs) and 95% confidence intervals (CIs) for lifetime history of depression per unit increase in Big Five personality trait scores among the N = 3065 participants of the DACH survey. Supplemental Table S3: Odds ratios and 95% confidence intervals for lifetime history of depression per unit increase in Big Five personality trait scores among the N = 3065 participants of the DACH survey stratified by gender. Supplemental Table S4: Odds ratios and 95% confidence intervals for lifetime history of depression per unit increase in Big Five personality trait scores among the N = 3065 participants of the DACH survey stratified by age. Supplemental Figure S1: Functional form of the observed relationships of age and the Big Five personality traits with depression risk, stratified by gender. Supplemental Figure S2: Functional form of the observed relationship of age and the Big Five personality traits with depression risk, stratified by age group. Supplemental Figure S3: Functional form of the predicted relationship of age and the Big Five personality traits with depression risk, stratified by gender (predictions based on Model 3*). Supplemental Figure S4: Functional form of the predicted relationship between depression risk and Big Five personality traits stratified by age group (predictions based on Model 3*).
