# Supplementary material for: The Relationship between Big Five Personality Traits and Depression in the German-Speaking D-A-CH Region Including an Investigation of Potential Moderators and Mediators
Source: Eur J Investig Health Psychol Educ. 2024 Jul 30;14(8):2157–74. doi: 10.3390/ejihpe14080144 (PMC11353565; doi:10.3390/ejihpe14080144)
Supplement: Supplementary file 1 [file ejihpe-14-00144-s001.zip › ejihpe-3092283-supplementary.pdf]

Supplemental Material to Strohmaier et al. "The relationship between Big Five personality traits and depression in the German speaking D-A-CH region including an investigation of potential moderators and mediators"

Supplemental Table S1. Distribution of Big Five personality among the entire study population as well as gender and age strata.

Variables [a]	Overall (n=3065)	Female (n = 1567)	Male (n=1498)	Aged 18-39 years (n=1022)	Aged 40-59 years (n=1138)	Aged 60+ years (n=905)
Neuroticism	11.48 (3.93)	12.22 (4.01)	10.70 (2.62)	12.62 (3.48)	11.51 (4.04)	10.14 (3.86)
Extroversion	13.09 (3.71)	13.24 (3.80)	12.93 (3.62)	12.80 (3.67)	13.08 (3.81)	13.43 (3.62)
Openness	13.87 (3.83)	14.03 (3.86)	13.70 (3.81)	14.11 (3.54)	13.81 (4.02)	13.67 (3.91)
Agreeableness	14.80 (3.18)	15.06 (3.16)	14.52 (3.18)	14.34 (3.01)	14.78 (3.22)	15.34 (3.24)
Conscientiousness	15.92 (3.32)	16.15 (3.30)	15.67 (3.31)	14.96 (3.22)	16.25 (3.29)	16.57 (3.21)

[a] Mean ± SD (all such values)

Supplemental Table S2. Odds ratios (ORs) and 95% confidence intervals (CIs) for life-time history of depression per unit increase in Big Five personality trait scores among the N=3, 065 participants of the DACH survey.

N= 3065<br>Case =507	Model 1[1]	Model 2[2]	Model 3[3]	Model 4[4]
	OR (95% CI)	OR (95% CI)	OR (95% CI)	OR (95% CI)
Neuroticism	1.23 (1.19 - 1.26)	1.22 (1.18 - 1.26)	1.21 (1.17 - 1.24)	1.14 (1.10 - 1.18)
Extroversion	0.94 (0.92 - 0.97)	0.98 (0.95 - 1.01)	0.98 (0.95 - 1.01)	0.97 (0.94 - 1.01)
Openness	1.03 (1.00 - 1.05)	1.05 (1.02 - 1.08)	1.04 (1.01 - 1.07)	1.03 (1.00 - 1.06)
Agreeableness	0.96 (0.93 - 0.99)	1.00 (0.97 - 1.04)	1.01 (0.97 - 1.05)	0.98 (0.94 - 1.02)
Conscientiousness	0.93 (0.91 - 0.96)	0.95 (0.91 - 0.98)	0.96 (0.92 - 0.99)	0.95 (0.91 - 0.98)

[1] Model 1: adjusted for gender and age (using restricted cubic splines with 5 knots)
[2] Model 2: additionally adjusted for all other Big Five personality traits
[3] Model 3: additionally adjusted for country of residence (Austria, Germany, Switzerland), migration history (first generation, second generation, no migration history), marital status (single, married or in a relationship, divorced, widowed), income (continuous), education (middle school, apprenticeship, high school, university degree), ethnicity (Caucasian, other), BMI (using restricted cubic splines with 4 knots), exercise (number of exercise units/week) , smoking status (never, former, current), employment (full-time, part-time, not currently employed, missing), night shift work (yes, no)
[4] Model 4: additionally adjusted for empathy score (restricted cubic spline with 3 knots), perspective taking (restricted cubic spline with 3 knots), optimism score (restricted cubic spline with 3 knots), work-life balance score, interpersonal trust score

Supplemental Table S3. Odds ratios and 95% confidence intervals for life-time history of depression per unit increase in Big Five personality trait scores among the N=3, 065 participants of the DACH survey stratified by gender

	Model 1[1]	Model 2[2]	Model 3[3]	Model 4[4]	p-Interaction
	OR (95% CI)	OR (95% CI)	OR (95% CI)	OR (95% CI)	
Males (n=1,498, cases =201)					
Neuroticism	1.24 (1.18 - 1.30)	1.24 (1.18 - 1.30)	1.21 (1.15 - 1.28)	1.14 (1.08 - 1.21)	
Extroversion	0.95 (0.91 - 0.99)	0.99 (0.94 - 1.04)	0.98 (0.93 - 1.03)	0.97 (0.92 - 1.03)	
Openness	1.04 (1.00 - 1.08)	1.05 (1.01 - 1.10)	1.05 (1.01 - 1.11)	1.04 (0.99 - 1.10)	
Agreeableness	0.95 (0.90 - 0.99)	1.01 (0.96 - 1.07)	1.02 (0.96 - 1.08)	1.00 (0.93 - 1.06)	
Conscientiousness	0.94 (0.90 - 0.98)	0.96 (0.91 - 1.02)	0.98 (0.92 - 1.04)	0.97 (0.92 - 1.03)	
Females (n=1,567, cases=306)					
Neuroticism	1.22 (1.18 - 1.27)	1.21 (1.17 - 1.26)	1.21 (1.16 - 1.26)	1.15 (1.10 - 1.20)	0.93
Extroversion	0.94 (0.91 - 0.98)	0.98 (0.94 - 1.02)	0.98 (0.94 - 1.02)	0.97 (0.93 - 1.02)	0.75
Openness	1.02 (0.99 - 1.06)	1.04 (1.00 - 1.08)	1.04 (1.00 - 1.08)	1.02 (0.98 - 1.07)	0.62
Agreeableness	0.97 (0.93 - 1.00)	1.00 (0.96 - 1.05)	1.01 (0.96 - 1.06)	0.97 (0.92 - 1.03)	0.33
Conscientiousness	0.93 (0.89 - 0.97)	0.93 (0.89 - 0.98)	0.94 (0.89 - 0.99)	0.92 (0.88 - 0.97)	0.21

[1] Model 1: adjusted for gender and age (using restricted cubic splines with 5 knots)

[2] Model 2: additionally adjusted for all other Big Five personality traits

[3] Model 3: additionally adjusted for country of residence (Austria, Germany, Switzerland), migration history (first generation, second generation, no migration history), marital status (single, married or in a relationship, divorced, widowed), income (continuous), education (middle school, apprenticeship, high school, university degree), ethnicity (Caucasian, other), BMI (using restricted cubic splines with 4 knots), exercise (number of exercise units/week) , smoking status (never, former, current), employment (full-time, part-time, not currently employed, missing), night shift work (yes, no)

[4] Model 4: additionally adjusted for empathy score (restricted cubic spline with 3 knots), perspective taking (restricted cubic spline with 3 knots), optimism score (restricted cubic spline with 3 knots), work-life balance score, interpersonal trust score

Supplemental Table S4. Odds ratios and 95% confidence intervals for life-time history of depression per unit increase in Big Five personality trait scores among the N=3, 065 participants of the DACH survey stratified by age

	Model 1[1]	Model 2[2]	Model 3[3]	Model 4[4]	p-Interaction
	OR (95% CI)	OR (95% CI)	OR (95% CI)	OR (95% CI)	
Aged 18 – 39 years (n = 1022, cases =158)					
Neuroticism	1.24 (1.18 - 1.32)	1.22 (1.15 - 1.30)	1.22 (1.15 - 1.29)	1.13 (1.05 - 1.21)	
Extroversion	0.93 (0.89 - 0.97)	0.97 (0.92 - 1.02)	0.96 (0.90 - 1.01)	0.95 (0.90 - 1.01)	
Openness	1.03 (0.98 - 1.08)	1.05 (1.00 - 1.11)	1.05 (0.99 - 1.12)	1.03 (0.96 - 1.10)	
Agreeableness	0.96 (0.91 - 1.02)	1.00 (0.94 - 1.07)	1.01 (0.94 - 1.08)	0.98 (0.90 - 1.06)	
Conscientiousness	0.93 (0.88 - 0.98)	0.94 (0.88 - 1.00)	0.95 (0.89 - 1.02)	0.96 (0.89 - 1.03)	
Aged 40 – 59 years (n= 1138, cases =240)					
Neuroticism	1.21 (1.16 - 1.26)	1.21 (1.16 - 1.26)	1.21 (1.15 - 1.26)	1.13 (1.07 - 1.20)	
Extroversion	0.96 (0.93 - 1.00)	1.00 (0.96 - 1.04)	1.00 (0.96 - 1.05)	0.99 (0.95 - 1.04)	
Openness	1.02 (0.98 - 1.05)	1.02 (0.98 - 1.06)	1.01 (0.96 - 1.05)	1.01 (0.97 - 1.06)	
Agreeableness	0.97 (0.93 - 1.01)	1.00 (0.95 - 1.06)	1.01 (0.96 - 1.07)	0.98 (0.92 - 1.04)	
Conscientiousness	0.96 (0.91 - 1.00)	0.98 (0.93 - 1.03)	0.98 (0.93 - 1.04)	0.97 (0.91 - 1.03)	
Aged 60+ years (n= 905, cases =109)					
Neuroticism	1.23 (1.16 - 1.30)	1.23 (1.16 - 1.30)	1.24 (1.17 - 1.32)	1.22 (1.13 - 1.31)	0.79
Extroversion	0.94 (0.89 - 1.00)	0.97 (0.91 - 1.03)	0.96 (0.90 - 1.02)	0.97 (0.91 - 1.04)	0.57
Openness	1.05 (1.00 - 1.11)	1.10 (1.04 - 1.17)	1.09 (1.03 - 1.17)	1.07 (1.00 - 1.15)	0.38
Agreeableness	0.94 (0.88 - 1.00)	1.01 (0.94 - 1.09)	1.03 (0.95 - 1.11)	1.01 (0.93 - 1.11)	0.94
Conscientiousness	0.90 (0.85 - 0.96)	0.90 (0.84 - 0.97)	0.90 (0.83 - 0.97)	0.88 (0.81 - 0.95)	0.55

[1] Model 1: adjusted for gender and age (using restricted cubic splines with 5 knots)
[2] Model 2: additionally adjusted for all other Big Five personality traits
[3] Model 3: additionally adjusted for country of residence (Austria, Germany, Switzerland), migration history (first generation, second generation, no migration history), marital status (single, married or in a relationship, divorced, widowed), income (continuous), education (middle school, apprenticeship, high school, university degree), ethnicity (Caucasian, other), BMI (using restricted cubic splines with 4 knots), exercise (number of exercise units/week) , smoking status (never, former, current), employment (full-time, part-time, not currently employed, missing), night shift work (yes, no)
[4] Model 4: additionally adjusted for empathy score (restricted cubic spline with 3 knots), perspective taking (restricted cubic spline with 3 knots), optimism score (restricted cubic spline with 3 knots), work-life balance score, interpersonal trust score

Supplemental Figure S1. Functional form of the observed relationships of age and the Big Five personality traits with depression risk, stratified by gender

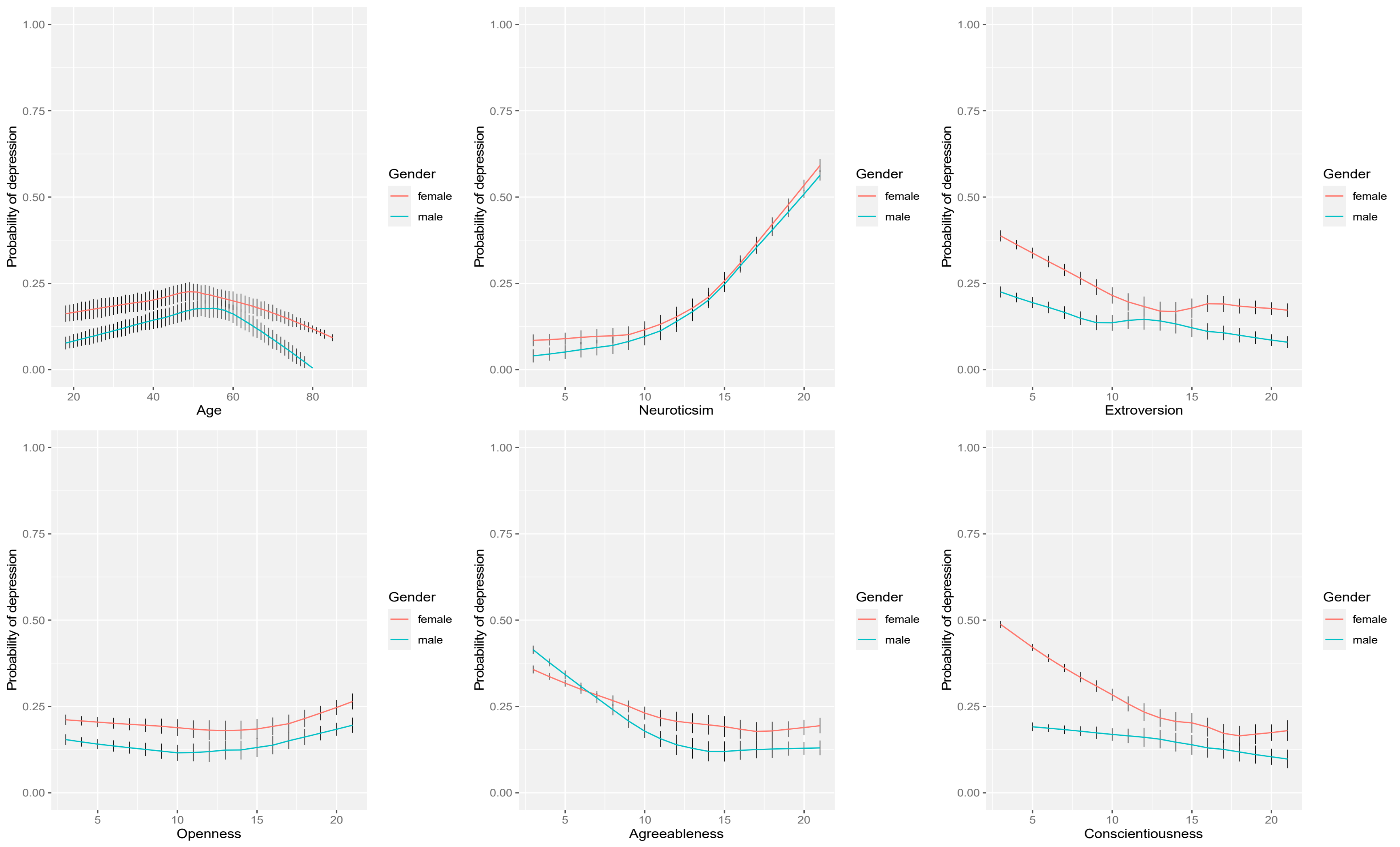

Supplemental Figure S2. Functional form of the observed relationship of age and the Big Five personality traits with depression risk, stratified by age group.

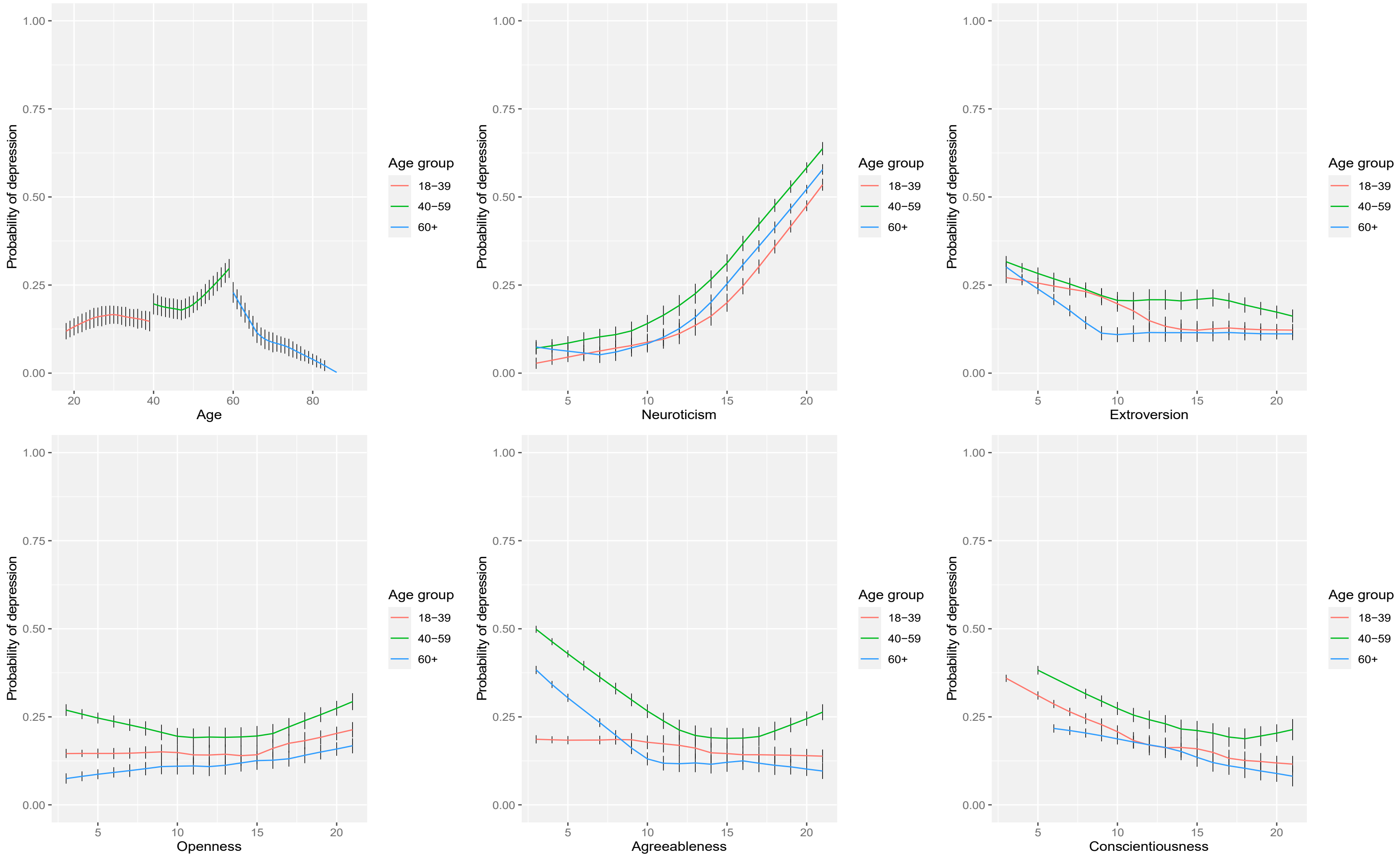

Supplemental Figure S3. Functional form of the predicted relationship of age and the Big Five personality traits with depression risk, stratified by gender (predictions based on Model 3*)

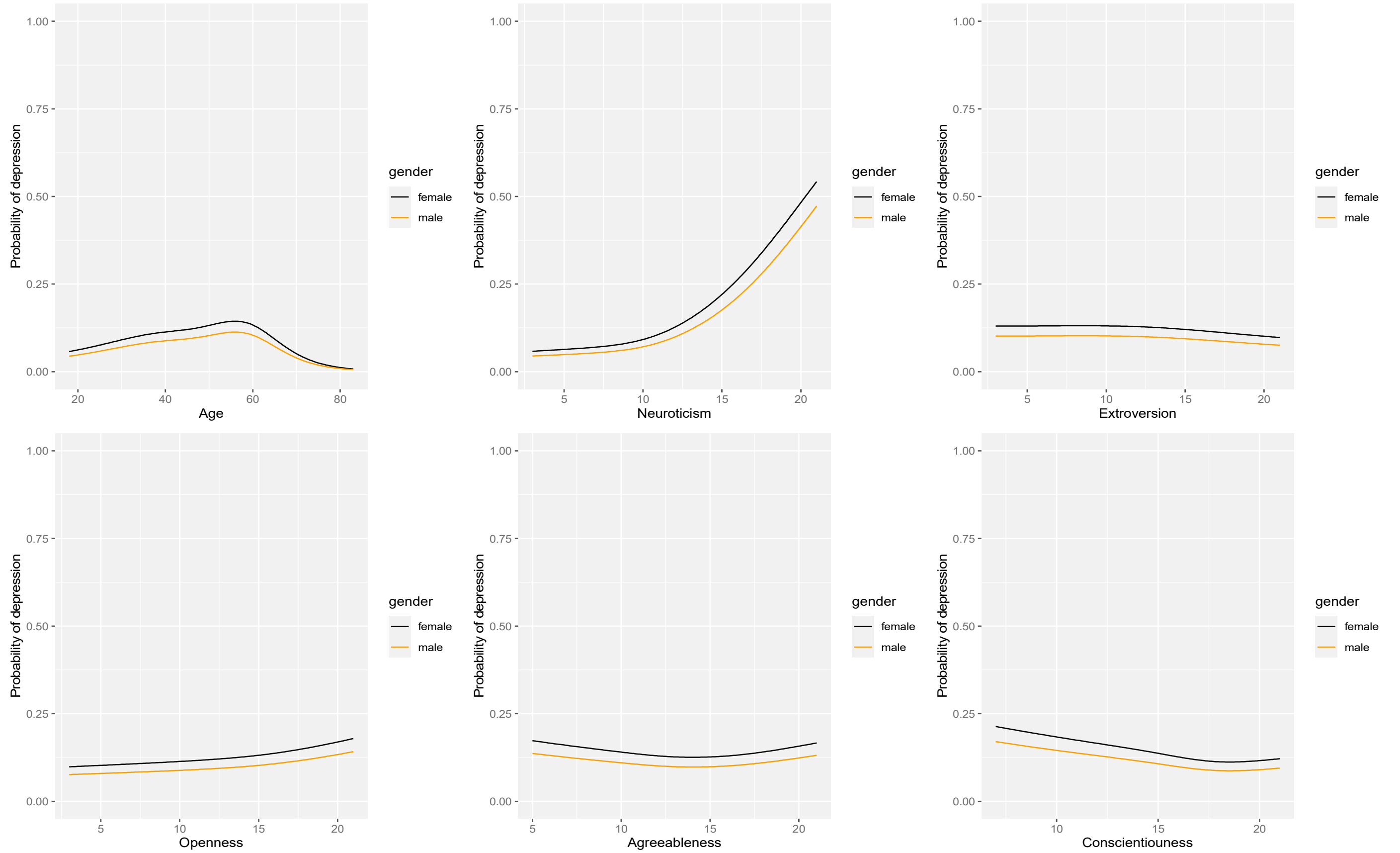

*Model 3: adjusted for gender and age (using restricted cubic splines with 5 knots), all other Big Five personality traits, country of residence (Austria, Germany, Switzerland), migration history (first generation, second generation, no migration history), marital status (single, married or in a relationship, divorced, widowed), income (continuous), education (middle school, apprenticeship, high school, university degree), ethnicity (Caucasian, other), BMI (using restricted cubic splines with 4 knots), exercise (number of exercise units/week) , smoking status (never, former, current), employment (full-time, part-time, not currently employed, missing), night shift work (yes, no)

Supplemental Figure S4. Functional form of the predicted relationship between depression risk and Big Five personality traits stratified by age group (predictions based on Model 3*)

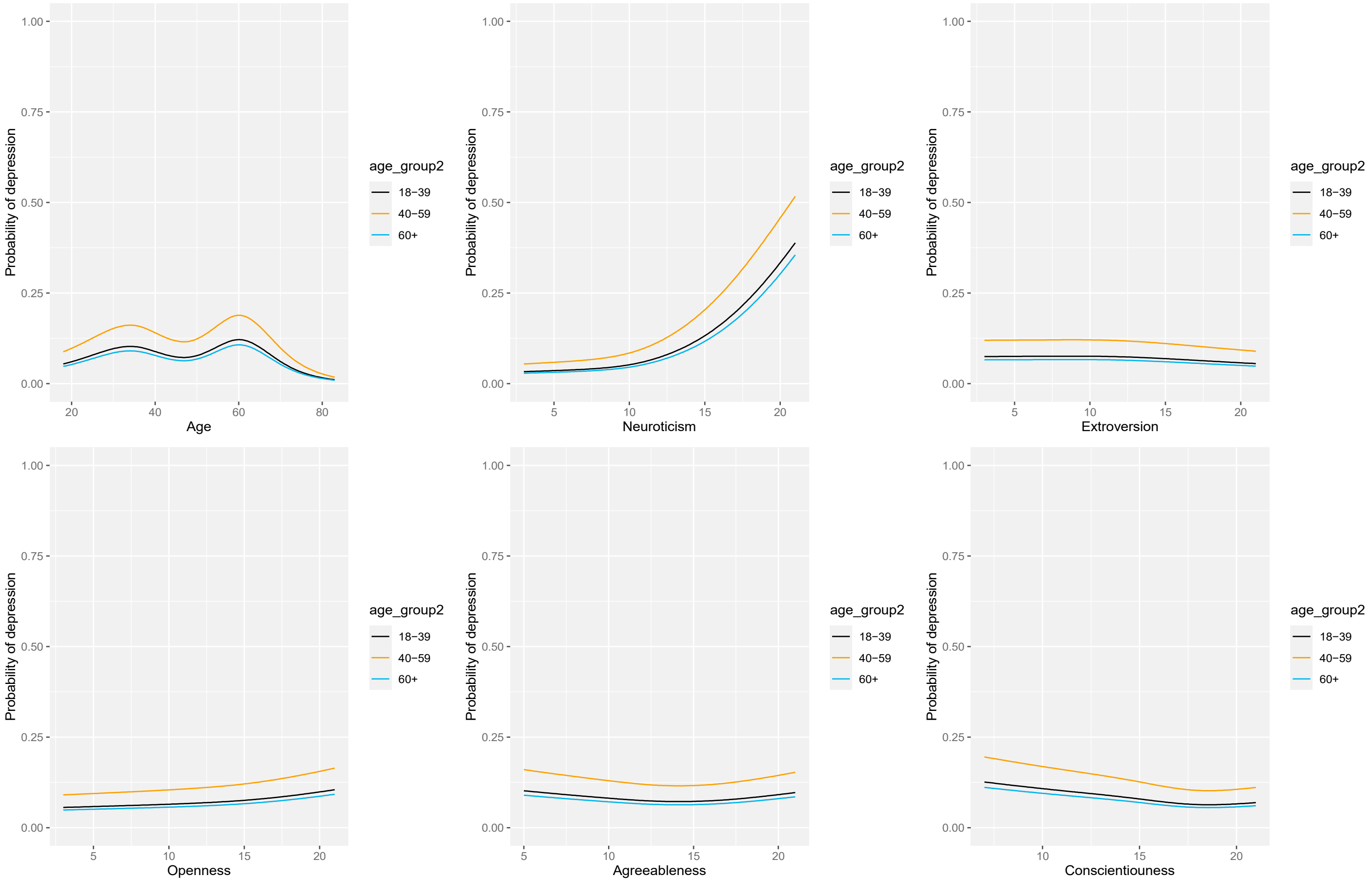

*Model 3: adjusted for gender and age (using restricted cubic splines with 5 knots), all other Big Five personality traits, country of residence (Austria, Germany, Switzerland), migration history (first generation, second generation, no migration history), marital status (single, married or in a relationship, divorced, widowed), income (continuous), education (middle school, apprenticeship, high school, university degree), ethnicity (Caucasian, other), BMI (using restricted cubic splines with 4 knots), exercise (number of exercise units/week) , smoking status (never, former, current), employment (full-time, part-time, not currently employed, missing), night shift work (yes, no)
